# Healthcare experience among patients with type 2 diabetes: A cross‐sectional survey using the IEXPAC tool

**DOI:** 10.1002/edm2.220

**Published:** 2021-02-15

**Authors:** Domingo Orozco‐Beltrán, Sara Artola‐Menéndez, Antonio Hormigo‐Pozo, Daniel Cararach‐Salami, Juan Luis Alonso‐Jerez, Epifanio Álvaro‐Grande, Covadonga Villabrille‐Arias, Francisco Javier de Toro‐Santos, María José Galindo‐Puerto, Ignacio Marín‐Jiménez, Antón Gómez‐García, Rocío Ledesma‐Rodriguez, Gonzalo Fernández, Karine Ferreira de Campos

**Affiliations:** ^1^ Clinical Medicine Department Miguel Hernandez University San Juan de Alicante Spain; ^2^ José Marvá Heath Care Center SED (Spanish Diabetes Society) Diabetes Group Madrid Spain; ^3^ San Andres‐Torcal Health Care Center Málaga Spain; ^4^ Les Corts Health Care Center Barcelona Spain; ^5^ Tejina Health Care Center Tenerife Spain; ^6^ Santutxu‐Solokoetxe Health Care Center Bilbao Spain; ^7^ Pumarín Health Care Center Oviedo Spain; ^8^ Rheumatology Department A Coruña University Hospital A Coruña Spain; ^9^ Internal Medicine Department Clinic University Hospital Valencia Spain; ^10^ Research Department SEISIDA (Spanish AIDS Multidisciplinary Society Madrid Spain; ^11^ Gastroenterology Department Hospital Universitario Gregorio Marañón Madrid Spain; ^12^ Medical Affairs Department Merck Sharp & Dohme Madrid Spain

**Keywords:** chronic disease, patient experience, patient survey, type 2 diabetes

## Abstract

**Aim:**

To assess the experience with health care among patients with type 2 diabetes (T2DM) and to evaluate patients’ demographic variables and healthcare‐related characteristics which may affect their experience.

**Methods:**

A cross‐sectional survey was delivered to T2DM adults. Patient experiences were assessed with the ‘Instrument for Evaluation of the Experience of Chronic Patients’ (IEXPAC) questionnaire, a validated 12‐item survey, which describes patient experience within the last 6 months (items 1–11) and hospitalization in the last 3 years (item 12), with possible scores ranging from 0 (worst) to 10 (best experience).

**Results:**

A total of 451 T2DM patients responded to the survey (response rate 72.3%; mean age 69.5 ± 10.1 years, 67.8% men). The mean overall IEXPAC score was 5.92 ± 1.80. Mean scores were higher for productive interactions (7.92 ± 2.15) and self‐management (7.08 ± 2.27) than for new relational model (1.72 ± 2.01). Only 32.8% of patients who had been hospitalized in the past 3 years reported having received a follow‐up call or visit after discharge. Multivariate analyses identified that regular follow‐up by the same physician and follow‐up by a nurse were associated with a better patient experience. Continuity of healthcare score was higher only in those patients requiring help from others.

**Conclusions:**

The areas of T2DM care which may need to be addressed to ensure better patient experience are use of the Internet, new technologies and social resources for patient information and interaction with healthcare professionals, closer follow‐up after hospitalization, and a comprehensive multidisciplinary approach with regular follow‐up by the same physician and a nurse.

## Graphical abstract

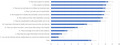

## 1 INTRODUCTION

Patient experience with health care is a key component for the provision of a patient‐centred healthcare model, as both clinical effectiveness and safety are correlated positively with patient experience.^1^ In patients with chronic conditions, a more positive patient experience is associated with improved care quality with the interaction of patients with healthcare professionals^2^ particularly general practitioners, being important for patient well‐being.^3^ Effective chronic illness management also depends on multidisciplinary care teams, including nurses and pharmacists, with clinical experience.^4^

Diabetes is a major public health problem that is approaching epidemic proportions globally. Annually, >3 million (5.2%) deaths are attributable to diabetes making it a leading cause of death worldwide.^5^ Diabetes is also associated with poor quality of life (QoL) and disability.^6^, ^7^ Despite being largely preventable, type 2 diabetes mellitus (T2DM) accounts for around 90% of diabetes cases, affecting 294.3 million people in 2017 and rising globally, and predicted to affect 394.2 million people by 2045.^8^ In Spain, the prevalence of T2DM is estimated to be 13.8% (with almost half of these cases being undiagnosed DM)^9^ and the direct health costs of diabetes are around 8% of total public health expenditures (€5.1 billion in 2009). The annual cost per diabetic patient averages close to €1,660 for direct costs and €916 for productivity losses.^10^ As a result, ascertaining healthcare experience in diabetic populations is important.

Previously, we have reported the outcomes of a survey to assess the experience of a diverse group of patients with four different chronic conditions (T2DM, human immunodeficiency virus infection, inflammatory bowel disease and rheumatic diseases) with health care using the Instrument to Evaluate the EXperience of PAtients with Chronic diseases (IEXPAC).^11^ IEXPAC is a validated questionnaire, developed in Spain, with several advantages over other available questionnaires (namely, focusing on the overall interaction of patients with the healthcare system and not with specific professionals, and the inclusion of a broader notion of integrated care, including social care, patients’ self‐management, new technological interventions and patients’ interactions with other patients).^12^ Herein, we focus on the cohort of patients with T2DM, with the objective of describing patient perception of health care, to identify the main areas for improvement and to assess potential variables affecting patient experience including demographic variables and healthcare‐related characteristics.

## 2 METHODS

This was a cross‐sectional study, where a survey was given to patients with T2DM with cardiovascular or renal complications, aged >18 years, receiving health care from primary care centres across eight Spanish Autonomous Communities: Andalusia, Asturias, Basque Country, Canary Islands, Castilla La Mancha, Catalonia, Madrid and Valencia. The first 13 consecutive patients attending each primary care centre who met inclusion criteria received a survey, which included the IEXPAC questionnaire as its focus, from 48 primary care physicians. Surveys were distributed and collected between May and September 2017. The study protocol, methodology and main outcomes for the overall population have been described previously.^11^ The main objectives of the current study were to describe patients’ experience with health care, to identify the main areas for improvement and to assess potential variables affecting patient experience among a population with T2DM. The study was reviewed and approved by the Clinical Investigation Ethics Committee of the Gregorio Marañón Hospital, Madrid, Spain. Patients provided written informed consent before entering the study.

### 2.1 Survey instrument

The survey mainly included the IEXPAC questionnaire plus additional multiple‐choice questions in order to provide information on patient demographics, healthcare and treatment‐related characteristics. The survey was drafted by expert physicians and reviewed and finally endorsed by the Spanish Diabetes Federation (FEDE) among other patients’ associations.

Details of the IEXPAC questionnaire have been published.^12^ The questionnaire was in Spanish. Briefly, IEXPAC is a self‐administered 12‐item questionnaire with patient responses made using a 5‐point Likert scale: always (score 10), mostly (7.5), sometimes (5), seldom (2.5) or never (0). An overall score is given by summing the scores of items 1–11, which describe patient experience within the last 6 months, ranging from 0 (worst experience) to 10 (best experience). Item 12, describing continuity of health care after hospitalization in the last 3 years, is reported separately.

Three factors are derived from IEXPAC items 1–11. Factor 1 (productive interactions) refers to the content and characteristics of interactions between patients and healthcare professionals and is the mean score of items 1, 2, 5 and 9. Factor 2 (new relational model) refers to new forms of patient interaction with the healthcare system through the Internet or with peers and is the mean score of items 3, 7 and 11. Factor 3 (patient self‐management) captures the ability of individuals to manage their own care and improve their well‐being based on healthcare professional‐mediated interventions and is the mean score of items 4, 6, 8 and 10.

### 2.2 Other variables measured

Beliefs about medication were determined using the Beliefs About Medicines Questionnaire (BMQ).^13^, ^14^ The BMQ evaluates an individual's opinion about medicines in general (abuse and damage) and about specific drugs for his/her disease (need and concern). This 10‐item questionnaire covers two domains—Necessity and Concerns, with five statements per domain. Patients respond on a 5‐point Likert scale, ranging from strongly disagree (scored 1) to strongly agree (scored 5). Scores are summed for each individual item, and total scores for the Necessity and Concerns domains (each ranging from 5 to 25) are also calculated. Higher scores in the Necessity and Concerns scales indicate stronger beliefs in the necessity for the prescribed medication and more concern about taking the medication, respectively. The overall BMQ score (presented in this study) is calculated as the difference between the Necessity Scale and Concerns Scale scores, with a possible range of −20 to 20. Higher overall scores indicate stronger beliefs.

A visual analogue scale (VAS) was used to determine health status. It is a psychometric response scale across a continuum of values that ranges from 0 (worst health status) to 100 (best health status).^15^

The Barthel scale was used to measure performance in activities of daily living. The Barthel disability index ranges from 0 to 100: 0–20: ‘total’ dependency; 21–60: ‘severe’ dependency; 61–90: ‘moderate’ dependency; 91–99: ‘slight’ dependency; 100: independent.^16^

### 2.3 Statistical analysis

This was an exploratory study with no formal hypothesis nor pre‐specified sample size. A conservative approach was adopted to calculate sample size based on a qualitative variable with an expected prevalence of 50%, 95% confidence interval and with 6% precision giving an initial calculated sample size of 267 patients, plus 15% of variables completed incorrectly (an additional 47 patients; total, 314 patients), and accounting for an expected response rate of approximately 50%, as found in other surveys handed to patients by clinical teams^17^, ^18^ to give a total sample size of 628 patients. Then, 48 primary care centres were selected to be representative of the Spanish population. As a result, it was calculated that at least 13 patients for each primary care centre were required to complete the sample size.

Descriptive information is displayed as mean and standard deviation for quantitative variables, and frequencies or percentages for qualitative variables. The results of the IEXPAC questionnaire were calculated as overall mean and standard deviation score and mean and standard deviation scores for Factors 1–3. The distribution of responses to each item was also displayed, as well as the mean score for each item.

The chi‐squared or Fisher exact tests were used for comparisons of proportions and the Student t test or analysis of variance used to compare continuous variables. Multiple linear regression models were used to assess different demographic and healthcare‐related variables influencing the IEXPAC overall score and individual factor scores. Beta coefficients with *p*‐values are shown (positive coefficients indicate higher IEXPAC experience scores). Given the overall descriptive nature of the results, no multiplicity adjustments were made and there was no imputation for missing data.

## 3 RESULTS

### 3.1 Description of the sample

In the overall study population, 1,618 patients completed the survey (response rate 65.4%), of which 451 patients had diabetes (27.9%) and were included in the final study analysis (response rate 72.3%). Patients’ demographic and healthcare‐related characteristics are presented in Table 1. The mean age of responders was 69.5 ± 10.1 years, 67.8% of patients were men and mean Barthel index was 92.7 ± 17.4. Only 4.6% were affiliated to a patients’ association, and almost half of patients (48.9%) had searched for information about health care from sources different to those provided by healthcare providers. For 4.4% of patients, follow‐up for health care was in a Spanish region different from their residing region. The mean number of different specialists visited in the last year, including general practitioners, was 4.0 ± 2.4, with the two most common being primary care physicians (90.9%) and cardiologists (46.6%). Only 66.8% of patients were generally followed‐up by the same physician and most patients (82.7%) received additional follow‐up by a nurse. Support from non‐healthcare workers (relatives, friends or caregivers) for patients’ health care was received by 45.2% of patients, and 56.1% of patients had been hospitalized at least once in the past 3 years. With regard to medications, patients were taking a mean of 6.5 ± 3.2 different pills daily (ie if someone took a medicine twice a day, the number of different medicines would be two), and 23.9% were receiving injectable medications.

**TABLE 1:** Patient demographics and healthcare‐related characteristics of patients who completed the survey (n = 451).

Parameter	Value
***Patient demographics***	
Age, mean, years	69.5 ± 10.1
Sex, Men, %	67.8
Educational level achieved, %	
Primary or no studies	50.8
Secondary, including vocational	28.8
University or further	20.4
Employment status, %	
Retired	65.9
Worker	16.3
Sick leave/disability	7.2
Household work	7.0
Unemployed	3.6
Barthel Index of Activities of Daily Living, Barthel ≤80, %	9.0
Barthel Index, mean	92.7 ± 17.4
Affiliated to patients’ association, %	4.6
Searched for information about health care from sources different to healthcare providers (ie Internet, media, etc.), %	48.9
***Healthcare‐related characteristics***	
Follow‐up for health care in a Spanish region different from the patient's main residence, %	4.4
Number of different specialists (including primary care) visited within the past year, mean (SD)	4.0 ± 2.4
0 specialists, %	2.2
1–2 specialists, %	25.5
3–4 specialists, %	40.1
≥5 specialists, %	32.2
Most common specialists visited in the last year, %	
Primary care physician	90.9
Cardiologist	46.6
Ophthalmologist	37.5
Traumatologist	22.8
Endocrinologist	22.0
Vascular surgeon	16.9
Pneumologist	16.6
Patient follow‐up usually performed by the same physician, %	
Generally, the same physician	66.8
Sometimes different	26.0
Frequently different	7.2
Additional follow‐up by a nurse, %	82.7
Number of visits to the emergency department within the last year, mean	1.4 ± 1.8
Proportion of patients attended in the emergency department within the last year, %	64.2
Support from others (relatives or friends, caregiver) for health care, %	45.2
Hospitalization within the past 3 years, %	56.1
***Treatment‐related characteristics***	
Number of medicines taken daily, mean	6.5 ± 3.2
0–4, %	31.3
5–7, %	33.4
≥ 8, %	35.3
Treated with subcutaneous or intravenous medications, %	23.9

### 3.2 IEXPAC responses and experience scores

**FIGURE 1:**
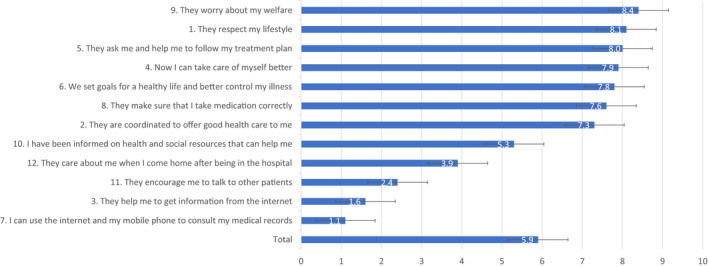
Mean scores for each IEXPAC item.

The mean overall IEXPAC score was 5.92 ± 1.80 (Figure 1 and Supplementary Table 1). Mean scores were higher for Factor 1 (Productive interactions score: 7.92 ± 2.15) and Factor 3 (Self‐management score: 7.08 ± 2.27) than for Factor 2 (New relational model score: 1.72 ± 2.01). The proportion of patients who responded ‘always’ or ‘mostly’ to items related to the Productive interactions score (items 1, 2, 5 and 9) was >70%. By contrast, regarding the New relational model score (items 3, 7 and 11), the majority of patients responded ‘seldom’ or ‘never’ to the 3 items. Regarding the Patient self‐management score (items 4, 6, 8 and 10), except for being informed on health and social resources, >70% of patients responded ‘always’ or ‘mostly’. Only 32.8% of patients who had been hospitalized in the past 3 years reported having received a follow‐up call or visit after discharge (Table 2).

**TABLE 2:** Patient responses (%) and mean scores for each IEXPAC item.

IEXPAC item	Patient responses (%)					Mean score
	Always	Mostly	Sometimes	Seldom	Never	
**1. They respect my lifestyle** The professionals who care of me listen to me and ask me about my needs, habits and preferences to adapt my treatment and care plan	51.2	30.7	12.2	3.2	2.7	8.1 ± 2.4
	81.9			5.9		
**2. They are coordinated to offer good health care to me** Health and social care services are coordinated to improve my well‐being and quality of life in my environment (family, neighbourhood, town).	44.1	29.6	11.0	5.2	10.1	7.3 ± 3.2
	73.7			15.3		
**3. They help me to get information from the Internet** The professionals who care for me inform me about trustful webpages and Internet forums that I can consult to know my disease better, its treatment and the consequences they may have on my life.	5.1	3.2	10.0	13.0	68.7	1.6 ± 2.8
	8.3			81.7		
**4. Now I can take care of myself better** I feel that my confidence in my ability to take care of myself, manage my health problems and keep my autonomy has improved.	43.4	34.9	17.5	1.9	2.4	7.9 ± 2.3
	78.3			4.3		
**5. They ask me and help me to follow my treatment plan** I regularly review adherence to my treatment and care plan with the professionals who care for me.	52.2	26.0	15.1	2.3	4.4	8.0 ± 2.7
	78.2			6.7		
**6. We set goals for a healthy life and better control my illness** I’ve been able to agree with the professionals who care for me on specific objectives regarding diet, physical exercise and medication to get better control of my health problems.	47.3	28.8	14.8	4.6	4.4	7.8 ± 2.7
	76.1			9.0		
**7. I can use the Internet and my mobile phone to consult my medical records** I can consult my clinical record, tests results, programmed visits and access to other services through the Internet or the mobile app of my health service.	3.8	3.3	6.4	7.1	79.5	1.1 ± 2.5
	7.1			86.6		
**8. They make sure that I take medication correctly** The professionals who care for me review with me all of the medication I take, how I take it and how it suits me.	51.4	21.6	13.8	5.7	7.6	7.6 ± 3.1
	73.0			13.3		
**9. They worry about my welfare** The professionals who care for me are concerned with my quality of life and I feel they are committed to my well‐being	59.4	24.4	11.2	2.7	2.3	8.4 ± 2.4
	83.8			5.0		
**10. I have been informed on health and social resources that can help me** The professionals who care for me inform me about health and social resources available in my neighbourhood or town that I can use to improve my health problems and take better care of myself.	26.7	15.6	22.9	12.1	22.7	5.3 ± 3.7
	42.3			34.8		
**11. They encourage me to talk to other patients** The professionals who care for me invite me to participate in patients groups to share information and experiences on how to care for ourselves and improve our health.	5.7	6.2	19.1	16.5	52.4	2.4 ± 3.0
	11.9			68.9		
**Respond to the following statement only if you have been admitted to the hospital in the last 3 years** **12. They care about me when I come home after being in the hospital** After hospital discharge, they have called or visited me at home to see how I was and what care I needed.	24.5	8.3	13.0	7.5	46.6	3.9 ± 4.2
	32.8			54.1		

‘Productive Interactions’ factor: items 1, 2, 5 and 9; ‘New Relational Model’ factor: items 3, 7 and 11; ‘Patient Self‐Management’ factor: items 4, 6, 8 and 10

IEXPAC experience scores stratified by different demographic and healthcare‐related variables are shown in Supplementary Tables 1 and 2. By bivariate analysis, overall IEXPAC and Productive interactions scores were independent of gender, age, follow‐up in a region different from the home region, Barthel index, number of specialists visited in the last year, follow‐up by a nurse, having help from others for health care, number of drugs, being treated with injectable drugs and educational level achieved. However, overall IEXPAC and productive interactions scores were higher in patients followed‐up by the same physician compared to follow‐up by different physicians. With regard to the new relational model, scores were greater in younger patients and among patients with a higher educational level and there was a trend in patients followed‐up in a region different from the home region, without significant differences in the other healthcare‐related variables. Patient self‐management scores were greater in those patients followed‐up by the same physician (*p* = 0.01) and by a nurse (*p* = 0.02), and there was a trend to higher scores in elderly patients (*p* = 0.05). No other significant differences were reported.

Continuity of healthcare score was higher only in those patients requiring help from others, with no significant differences in the other healthcare‐related variables.

### 3.3 Multivariate analysis

Results of multiple linear regression analyses are shown in Table 3. Factors associated with higher overall IEXPAC score (therefore indicating a better experience) were being followed regularly by the same physician (*p* < 0.001) and receiving additional follow‐up by a nurse (*p* = 0.046). These variables were also associated with better self‐management scores (*p* < 0.001 and *p* = 0.003, respectively). Follow‐up by the same physician was also associated with higher productive interactions scores (*p* < 0.001) and higher item 12 scores (continuity of health care after hospitalization) (*p* = 0.01). Regarding the new relational model score, it was lower with increasing patients’ age (*p* = 0.001).

**TABLE 3:** Multivariate analysis: multiple regression analyses for the overall IEXPAC experience score and for Factors 1–3 and continuity of health care.

Parameter	Overall IEXPAC experience score		Productive Interactions (Factor 1)		New Relational Model (Factor 2)		Patient Self‐Management (Factor 3)		Continuity of health care after hospital discharge	
	Beta coeff (SD)	*p*	Beta coeff (SD)	*p*	Beta coeff (SD)	*p*	Beta coeff (SD)	*p*	Beta coeff (SD)	*p*
Sex (women *vs*. men)	0.00 ± 0.23	0.99	0.15 ± 0.28	0.60	−0.16 ± 0.27	0.54	0.01 ± 0.29	0.98	0.65 ± 0.72	0.37
Age (per year of increment)	−0.01 ± 0.01	0.40	0.01 ± 0.01	0.60	−0.04 ± 0.01*	**0.001**	0.00 ± 0.01	0.84	0.02 ± 0.03	0.51
Follow‐up in a region different from home region (*vs*. same region)	−0.41 ± 0.57	0.48	−0.12 ± 0.68	0.86	−0.51 ± 0.68	0.45	−0.55 ± 0.75	0.46	−1.92 ± 1.53	0.21
Barthel Index >80	−0.01 ± 0.01	0.23	−0.01 ± 0.01	0.23	−0.01 ± 0.01	0.43	−0.01 ± 0.01	0.39	−0.03 ± 0.02	0.11
Number of specialists visited in the last year (per unit of increment)	0.01 ± 0.05	0.79	0.02 ± 0.06	0.71	0.00 ± 0.62	0.95	0.03 ± 0.07	0.65	0.11 ± 0.16	0.49
Follow‐up by the same physician (*vs*. different)	−0.93 ± 0.24	< 0.001	−1.27 ± 0.28	< 0.001	−0.25 ± 0.27	0.36	−1.00 ± 0.29	0.001	−1.74 ± 0.69	0.01
Follow‐up by a nurse (*vs*. no nurse follow‐ up)	0.55 ± 0.27	0.046	0.46 ± 0.33	0.16	0.02 ± 0.31	0.96	1.01 ± 0.34	0.003	0.74 ± 0.83	0.37
Having help from others for health care (*vs*. only self‐care)	−0.31 ± 0.23	0.89	−0.09 ± 0.28	0.74	0.12 ± 0.27	0.65	−0.60 ± 0.29	0.84	1.19 ± 0.69	0.09
Number of different medicines (per unit of increment)	−0.21 ± 0.04	0.58	−0.02 ± 0.05	0.63	−0.00 ± 0.04	0.93	−0.02 ± 0.05	0.68	0.04 ± 0.11	0.73
Being treated with SC/IV drugs (*vs*. no SC/IV treatment)	−0,25 ± 0.26	0.33	−0.27 ± 0.31	0.39	0.01 ± 0.30	0.98	−0.49 ± 0.33	0.13	0.81 ± 0.79	0.31

Beta coeff, Beta coefficient; IV, intravenous; SC, subcutaneous;

* Denotes a linear trend. Positive coefficients indicate higher IEXPAC experience scores.

### 3.4 BMQ and Health Status VAS scores

Mean overall scores for the BMQ and Health Status VAS were 6.38 ± 5.87 and 66.96 ± 17.12, respectively (both slightly above the average for each respective scale).

## 4 DISCUSSION

Improving healthcare experience among patients with chronic conditions, such as T2DM, should be considered as a therapeutic goal, as it is associated with higher clinical effectiveness and safety.^1^ Using the IEXPAC tool^12^ this study described the experience of T2DM patients with the health care received, identifying positive aspects of patient experience but also areas for improvement. As outlined previously, although there are other questionnaires that assess the experience of chronic patients, the IEXPAC scale may provide a more complete approach about the experience of these patients with health care.^12^, ^19^, ^20^ Therefore, the associations found in this study between better IEXPAC scores and some healthcare‐related variables may be very relevant for promoting changes from the healthcare organization perspective.

The survey was based on 451 responding patients with T2DM (mean age 69.5 years; mean number of medications 6.5; mean VAS 67). As a result, patients who responded to this survey were elderly, polymedicated and with some impairment in health status. This is in line with previous studies that have shown that, in Spain, patients with diabetes have many comorbidities and a reduced QoL.^21^ Thus, patients included in our study are likely to be representative of the Spanish population with T2DM.

Overall, patient responses were much more positive for Productive interactions score and Self‐management factors than for the New relational model factor: most patients (>70%) provided positive responses (‘always/mostly’) for 7 of the 8 items in those two factors. The exception was item 10 (patient information on health and social resources), where only 42% of patients provided positive responses. This means that patients consider that they are not sufficiently informed about new technologies and the ways in which they can access information about their disease, diet, lifestyle and use of social resources (ie local or online patient associations). In fact, half of all patients declared searching for information via sources different to healthcare providers in the last 6 months. Of note, the time spent and the individualization of education promote better long‐term diabetes control.^22^ In addition, patient's perception about the number of times physicians demand information about patient's preferences is inferior to physicians’ perceptions.^23^ and approximately 40% of patients with T2DM in Spain are not satisfied with information received about the condition.^24^ However, despite the fact that use of the Internet and other sources of information by patients and healthcare professionals have been associated with clinical improvements in the management of patients with T2DM, they are markedly underused in clinical practice.^25^ In addition, the use of digital resources, such as telemedicine or specific applications, may improve outcomes in diabetes, including medication adherence, and patient satisfaction.^26^ In summary, physicians should promote the use of the Internet and digital resources to improve the management and healthcare experience in patients with T2DM. However, the promotion of the Internet use should always initially be coached or guided by professionals.

Another interesting point was the low percentage of patients participating in a patient association (<5%). Promoting the use of social resources and interactions between patients to improve T2DM management and outcomes is another area for improvement. Thus, it has been reported that group activities, including a structured patient‐to‐patient telephone intervention, focusing on individuals with T2DM may improve lifestyle and self‐management behaviours.^27^ Consequently, in patients with chronic conditions, such as diabetes, receiving support from other patients may have a positive impact on healthcare experience.

In addition, only about one‐third of patients who had been hospitalized reported having received a follow‐up call or visit following discharge, while >55% of all patients had been hospitalized in the past 3 years. As the prevalence of diabetes is continuously increasing, diabetes‐related hospitalizations are progressively more common, increasing healthcare costs. Ensuring good continuity of care after hospital discharge through a multi‐sectoral approach is mandatory in order to avoid early rehospitalizations and complications.^10^

Both bivariate and multivariate analyses showed that regular follow‐up by the same physician and follow‐up by a nurse were associated with a better patient experience. Following complex self‐care recommendations to improve diabetes management (ie diet, physical activity, glucose control and medications) requires a good physician‐patient relationship, based on emotional links and interpersonal trust.^28^ In this context, regular follow‐up by the same physician is desirable to improve patient experience. On the other hand, as our study showed, nurses play a key role in the care of diabetic patients.^29^ These data suggest the importance of building good patient‐healthcare professional relationships, particularly with regular follow‐up by the same physician, which may aid communication as well as a multidisciplinary approach, combining both physicians and nurse care in order to improve patients’ experience and a better healthcare model.

Additionally, this study shows that the IEXPAC survey might be a very useful tool to identify and achieve patient‐centred healthcare goals in the management of T2DM, as promoted by the recent American Diabetes Association and the European Association for the Study of Diabetes Guidelines.^30^ while facilitating comprehensive improvements in social care and long‐term healthcare quality.

The limitations of this study have been described previously.^11^ Since this was an anonymous survey, the profiles of patients who did not return the surveys were not known. Additionally, in general, these types of voluntary surveys tend to generate responses more frequently from motivated patients or patients who are particularly worried about their health. The consecutive inclusion of patients reduces selection bias but does not eliminate such bias. Also, study outcomes refer to specific patients’ profiles and do not account for individual differences in patient health literacy. There is an overwhelming amount of information available from various sources about diabetes, and the appropriate use of this information can depends on the health literacy of patients. Therefore, the specific outcomes obtained in this population deserve future study. Although the multivariate models explained only a small part of the variability, factors were identified that, if corrected, have the potential to improve healthcare quality and patient experience. Finally, definitive data are lacking about whether IEXPAC score improvements are linked directly with improvements in clinical effectiveness and health related QoL.

In conclusion, this study identified areas with the potential to improve T2DM patients’ experience if properly addressed, such as patient interaction with healthcare professionals via the Internet or with peers, provision of patient information on health and social resources, including the use of new technologies, and closer follow‐up after hospital discharge. Additionally, the study highlights the importance of patient‐healthcare professional relationships and the need for a comprehensive multidisciplinary approach, demonstrating that the engagement of nurses is crucial in the management of T2DM.

### 4.1 Informed consent

As agreed by the Clinical Investigation Ethics Committee, the voluntary return of completed questionnaires was taken as implied consent to participate in the study. No clinical data were collected in this study.

### 4.2 What's new?

- Improving healthcare experience among patients with chronic conditions (eg type 2 diabetes [T2DM]) may be considered a therapeutic goal as it is associated with better clinical effectiveness and safety.
- Using the IEXPAC tool in 451 patients with T2DM, we identified positive aspects of patient experience, regarding productive interactions, and self‐management score, but not for the new relational model. Being followed regularly by the same physician and receiving additional follow‐up by a nurse were associated with a better experience.
- Improvement areas of T2DM care to ensure better patient experience may include the use of the Internet, new technologies and social resources for patient information and interaction with healthcare professionals, closer follow‐up after hospitalization and a comprehensive multidisciplinary approach with regular follow‐up by the same physician and a nurse.

## CONFLICTS OF INTERESTS

DOB received payment for lectures including services on speakers’ bureaus from MSD, Sanofi, Novo Nordisk, Lilly.

MJGP has been consultant to Gilead and Janssen, received or have grants pending to Janssen, received payment for lectures including service on speakers’ bureaus from Gilead, Janssen, MSD, AbbVie, received payment for development of educational presentations from MSD, Janssen, Gilead.

IMJ has been board membership, consultant and received payment for lectures including service on speakers’ bureaus to AbbVie, Chiesi, FAES Farma, Falk‐Pharma, Ferring, Gebro Pharma, Hospira, Janssen, MSD, Otsuka Pharmaceutical, Pfizer, Shire, Takeda, Tillots and UCB Pharma.

AGG, RLR, GF and KFC are full‐time employees at Merck Sharp & Dohme Spain.

The other authors declare no conflict of interest related to this manuscript.

## AUTHORS’ CONTRIBUTIONS

GF, AGG, DOB, FJTS, MJGP and IMJ conceived and designed the study.

AGG and GF planned the study implementation.

KFC planned and coordinated the study implementation.

SAM, AHP, DCS, JLAJ, EAG and CVA substantially helped to collect the data.

DOB and KFC interpreted the results.

KFC wrote sections of the initial draft.

DOB, AGG, RLR, GF and KFC provided substantive suggestions for revision and critically reviewed subsequent iterations of the manuscript.

All the authors reviewed and approved final version of the paper.

## ETHICAL APPROVAL

The study was reviewed and approved by the Clinical Investigation Ethics Committee of the Gregorio Marañón Hospital, Madrid, Spain. Study documentation included printed instructions and information for patients regarding the anonymous nature of the survey and aggregated data processing, which ensured that patient identification was not possible.

## Supporting information

Table S1‐S2

## Data Availability

The data that support the findings of this study are available from the corresponding author upon reasonable request.

## References

[1] Doyle C , Lennox L , Bell D . A systematic review of evidence on the links between patient experience and clinical safety and effectiveness. BMJ Open. 2013;3:e001570. 10.1136/bmjopen-2012-001570 PMC3549241 23293244

[2] Cramm JM , Nieboer AP . High‐quality chronic care delivery improves experiences of chronically ill patients receiving care. Int J Qual Health Care. 2013;25:689‐695. 24123243 10.1093/intqhc/mzt065 PMC3842124

[3] Cramm JM , Nieboer AP . The importance of productive patient‐professional interaction for the well‐being of chronically ill patients. Qual Life Res. 2015;24:897‐903. 25267102 10.1007/s11136-014-0813-6 PMC4366564

[4] Wagner EH . The role of patient care teams in chronic disease management. BMJ. 2000;320:569‐572. 10688568 10.1136/bmj.320.7234.569 PMC1117605

[5] WHO . The top 10 causes of death. World Heath Organ WHO [Internet]. 2017 [cited 2018 Feb 12]; Available from: http://www.who.int/mediacentre/factsheets/fs310/en/

[6] Gretebeck KA , Blaum CS , Moore T , et al. Functional exercise improves mobility performance in older adults with type 2 diabetes: a randomized controlled trial. J Phys Act Health. 2019;16:461‐469. 31122111 10.1123/jpah.2018-0240

[7] Rossi MC , Nicolucci A , Ozzello A , et al. Impact of severe and symptomatic hypoglycemia on quality of life and fear of hypoglycemia in type 1 and type 2 diabetes. Results of the Hypos‐1 observational study. Nutr Metab Cardiovasc Dis. 2019;29:736‐743. 31153746 10.1016/j.numecd.2019.04.009

[8] International Diabetes Federation . IDF Diabetes Atlas. 8th edn. Brussels, Belgium: International Diabetes Federation; 2017.

[9] Soriguer F , Goday A , Bosch‐Comas A , et al. Prevalence of diabetes mellitus and impaired glucose regulation in Spain: the Di@bet.es Study. Diabetologia. 2012;55:88‐93. 21987347 10.1007/s00125-011-2336-9 PMC3228950

[10] Lopez‐Bastida J , Boronat M , Moreno JO , Schurer W . Costs, outcomes and challenges for diabetes care in Spain. Global Health. 2013;9:17. 23635075 10.1186/1744-8603-9-17 PMC3658938

[11] Orozco‐Beltrán D , de Toro J , Galindo MJ , et al. Healthcare experience and their relationship with demographic, disease and healthcare‐related variables: a cross‐sectional survey of patients with chronic diseases using the IEXPAC scale. Patient. 2019;12:307‐317. 30430456 10.1007/s40271-018-0345-1 PMC6525115

[12] Mira JJ , Nuño‐Solinís R , Guilabert‐Mora M , et al. Development and validation of an instrument for assessing patient experience of chronic illness care. Int J Integr Care. 2016;16:13. 10.5334/ijic.2443 PMC5350641 28435422

[13] Beléndez‐Vázquez M , Hernández‐Mijares A , Horne R . Evaluación de las creencias sobre el tratamiento: validez y fiabilidad de la versión española del Beliefs about Medicines Questionnaire. Int J Clin Health Psychol. 2007;7:767‐779.

[14] Horne R , Weinman J , Hankins M . The beliefs about medicines questionnaire: the development and evaluation of a new method for assessing the cognitive representation of medication. Psychol Health. 1999;14:1‐24.

[15] Voutilainen A , Pitkäaho T , Kvist T , Vehviläinen‐Julkunen K . How to ask about patient satisfaction? The visual analogue scale is less vulnerable to confounding factors and ceiling effect than a symmetric Likert scale. J Adv Nurs. 2016;72:946‐957. 26689434 10.1111/jan.12875

[16] Prodinger B , O'Connor RJ , Stucki G , Tennant A . Establishing score equivalence of the Functional Independence Measure motor scale and the Barthel Index, utilising the International Classification of Functioning, Disability and Health and Rasch measurement theory. J Rehabil Med. 2017;49:416‐422. 28471470 10.2340/16501977-2225

[17] Bos‐Touwen I , Schuurmans M , Monninkhof EM , et al. Patient and disease characteristics associated with activation for self‐management in patients with diabetes, chronic obstructive pulmonary disease, chronic heart failure and chronic renal disease: a cross‐sectional survey study. PLoS One. 2015;10:e0126400. 25950517 10.1371/journal.pone.0126400 PMC4423990

[18] Marín‐Jiménez I , Casellas F , Cortés X , et al. The experience of inflammatory bowel disease patients with healthcare: a survey with the IEXPAC instrument. Medicine. 2019;98:e15044. 30946348 10.1097/MD.0000000000015044 PMC6456160

[19] Glasgow RE , Wagner EH , Schaefer J , Mahoney LD , Reid RJ , Greene SM . Development and validation of the Patient Assessment of Chronic Illness Care (PACIC). Med Care. 2005;43:436‐444. 15838407 10.1097/01.mlr.0000160375.47920.8c

[20] Singer SJ , Friedberg MW , Kiang MV , Dunn T , Kuhn DM . Development and preliminary validation of the patient perceptions of integrated care survey. Med Care Res Rev. 2013;70:143‐164. 23161612 10.1177/1077558712465654

[21] García‐Soidán FJ , Villoro R , Merino M , Hidalgo‐Vega Á , Hernando‐Martín T , González‐Martín‐Moro B . Health status, quality of life, and use of healthcare resources by patients with diabetes mellitus in Spain. Semergen. 2017;43:416‐424. 27445223 10.1016/j.semerg.2016.06.004

[22] García‐Donaire JA , Franch‐Nadal J , Rodríguez‐Fortúnez P , Labrador‐Barba E , Orera‐Peña ML , Rodríguez de Miguel M . Epidemiological multicentre study on the education provided to patients with type 2 diabetes mellitus in the Spanish Health Care System. The Forma2 study. Semergen. 2018;44:5‐12. 28511878 10.1016/j.semerg.2017.01.008

[23] Franch‐Nadal J , Labrador Barba E , Gómez‐García MC , Buil‐Cosiales P , Millaruelo JM , Peña ML . Patient‐reported outcomes in type 2 diabetes mellitus: patients’ and primary care physicians’ perspectives in the Spanish health care system. Patient Prefer Adherence. 2015;9:1413‐1422. 26504375 10.2147/PPA.S87005 PMC4603711

[24] Orera Peña ML , Franch Nadal J , Labrador Barba E , Rodríguez FP .Level of patient satisfaction with information received at infirmary consultation on the treatment of type 2 mellitus diabetes in Spain. REFLEJA2 Study. 5th Congress on Nursing Research of Ibero‐American and Portuguese‐speaking Countries, At Coimbra (Portugal) 6–8 June 2016: A7960.

[25] Hansel B , Giral P , Gambotti L , et al. A fully automated web‐based program improves lifestyle habits and HbA1c in patients with type 2 diabetes and abdominal obesity: randomized trial of patient E‐coaching nutritional support (The ANODE Study). J Med Internet Res. 2017;19:e360. 29117929 10.2196/jmir.7947 PMC5700402

[26] Orozco‐Beltran D , Sánchez‐Molla M , Sanchez JJ , Mira JJ . ValCrònic research group. Telemedicine in primary care for patients with chronic conditions: the valcrònic quasi‐experimental study. J Med Internet Res. 2017;19:e400. 29246881 10.2196/jmir.7677 PMC5747596

[27] Jiang YY , Zhang XX , Mao F , Dong WL , Dong JQ . The impact evaluation of a community‐based intervention supporting type 2 diabetes mellitus patients in their self‐management of the disease. Zhonghua Yu Fang Yi Xue Za Zhi. 2019;53:206‐211. 30744298 10.3760/cma.j.issn.0253-9624.2019.02.016

[28] Beverly EA , Worley MF , Court AB , Prokopakis KE , Ivanov NN . Patient‐physician communication and diabetes self‐care. J Clin Outcomes Manag. 2016;23:509‐518.

[29] Mulder BC , Lokhorst AM , Rutten GE , van Woerkum CM . Effective nurse communication with type 2 diabetes patients: a review. West J Nurs Res. 2015;37:1100‐1131. 24757047 10.1177/0193945914531077

[30] Davies MJ , D’Alessio DA , Fradkin J , et al. A Consensus Report by the American Diabetes Association (ADA) and the European Association for the Study of Diabetes (EASD). Diabetes Care. 2018;2018(41):2669‐2701. 10.2337/dci18-0033 PMC6245208 30291106

