# Supplementary material for: Healthcare experience among patients with type 2 diabetes: A cross‐sectional survey using the IEXPAC tool
Source: Endocrinol Diabetes Metab. 2021 Feb 15;4(2):e00220. doi: 10.1002/edm2.220 (PMC8029499; doi:10.1002/edm2.220)
Supplement: Supplementary file 1 — Table S1‐S2 [file EDM2-4-e00220-s001.docx]

**Supplementary material, Table 1. IEXPAC experience scores: overall scores and scores for the different factors (bivariate analysis)**

|  | **All patients (n = 451)** | **Gender** | | | **Age (P25: 63.75 years; P50: 70.00 years; P75: 77.00 years)** | | | | | **Follow-up in a region different from home region** | | | **Barthel Index** | | | **Number of specialists visited in the last year** | | | |
| --- | --- | --- | --- | --- | --- | --- | --- | --- | --- | --- | --- | --- | --- | --- | --- | --- | --- | --- | --- |
|  |  | **Men** | **Women** | **P** | **1Q** | **2Q** | **3Q** | **4Q** | **P** | **Yes** | **No** | **P** | **0-80** | **>80** | **P** | **0-2** | **3-4** | **>4** | **P** |
| Overall IEXPAC experience score | 5.92±1.80 | 5.94±1.86 | 5.90±1.67 | 0.84 | 6.11±1.78 | 5.69± 1.88 | 5.88±1.85 | 6.10±1.61 | 0.90 | 6.58±2.20 | 5.91±1.79 | 0.16 | 5.90±1.59 | 5.97±1.78 | 0.84 | 6.15±1.90 | 5.82±1.81 | 5.90±1.65 | 0.31 |
| Productive interactions (factor 1) | 7.92±2.15 | 7.90±2.21 | 7.96±2.02 | 0.80 | 7.86±2.08 | 7.72±2.21 | 7.90±2.26 | 8.29±1.91 | 0.15 | 8.16±2.20 | 7.93±2.14 | 0.66 | 7.90±1.77 | 7.98±2.10 | 0.84 | 8.26±2.28 | 7.77±2.09 | 7.89±2.06 | 0.19 |
| New relational model (factor 2) | 1.72±2.01 | 1.80±2.08 | 1.54±1.84 | 0.26 | 2.46±2.31 | 1.60±2.07 | 1.41±1.63 | 1.28±1.64 | **<0.001** | 2.74±2.90 | 1.67±1.97 | 0.05 | 1.77±1.98 | 1.70±2.00 | 0.85 | 1.56±1.89 | 1.89±2.13 | 1.58±1.90 | 0.97 |
| Patient self-management (factor 3) | 7.08±2.27 | 7.07±2.35 | 7.10±2.10 | 0.91 | 6.96±2.07 | 6.77±2.35 | 7.19±2.39 | 7.54±2.13 | 0.05 | 8.01±2.67 | 7.05±2.24 | 0.09 | 6.91±1.81 | 7.11±2.27 | 0.62 | 7.39±2.52 | 6.80±2.22 | 7.18±2.04 | 0.55 |
| Continuity of healthcare after hospital discharge | 3.94±4.22 | 3.76±4.24 | 4.37±4.17 | 0.30 | 4.25±4.17 | 3.82±4.09 | 4.10±4.57 | 4.59±4.22 | 0.60 | 5.00±4.47 | 3.89±4.16 | 0.39 | 5.08±4.48 | 3.77±4.15 | 0.11 | 4.04±4.09 | 4.23±4.38 | 3.54±4.10 | 0.39 |

|  | **Follow-up by the same physician** | | | **Follow-up by a nurse** | | | **Having** **help from others for healthcare** | | | **Number of different medicines** | | | | **Being treated with SC/IV drugs** | | | **Educational level achieved** | | | |
| --- | --- | --- | --- | --- | --- | --- | --- | --- | --- | --- | --- | --- | --- | --- | --- | --- | --- | --- | --- | --- |
|  | **Yes** | **No** | **P** | **Yes** | **No** | **P** | **Yes** | **No** | **P** | **0-4** | **5-7** | **>7** | **P** | **Yes** | **No** | **P** | **1** | **2** | **3** | **P** |
| Overall IEXPAC experience score | 6.13±1.74 | 5.36±1.78 | **<0.001** | 5.97±1.77 | 5.61±1.91 | 0.12 | 5.84±1.71 | 5.94±1.85 | 0.62 | 5.95±2.03 | 5.93±1.60 | 5.71±1.79 | 0.30 | 6.14±1.63 | 5.83±1.82 | 0.15 | 5.89±1.71 | 5.93±2.02 | 6.04±1.70 | 0.53 |
| Productive interactions (factor 1) | 8.23±2.05 | 7.17±2.17 | **<0.001** | 7.97±2.13 | 7.69±2.22 | 0.30 | 7.91±2.08 | 7.92±2.19 | 0.96 | 8.02±2.38 | 7.93±1.95 | 7.65±2.19 | 0.18 | 8.17±1.93 | 7.86±2.17 | 0.23 | 7.93±2.06 | 7.84±2.39 | 8.03±2.06 | 0.85 |
| New relational model (factor 2) | 1.72±2.05 | 1.51±1.77 | 0.34 | 1.69±1.98 | 1.59±1.94 | 0.68 | 1.61±1.82 | 1.69±2.07 | 0.72 | 1.70±2.23 | 1.63±1.86 | 1.67±1.89 | 0.91 | 1.69±1.96 | 1.66±1.97 | 0.93 | 1.42±1.77 | 1.92±2.22 | 2.19±2.16 | **<0.01** |
| Patient self-management (factor 3) | 7.29±2.16 | 6.48±2.37 | **0.01** | 7.20±2.16 | 6.49±2.59 | **0.02** | 7.03±2.13 | 7.08±2.36 | 0.82 | 6.93±2.47 | 7.06±2.10 | 6.95±2.25 | 0.97 | 7.45±2.12 | 6.93±2.31 | 0.06 | 7.19±2.22 | 7.06±2.44 | 6.90±2.14 | 0.34 |
| Continuity of healthcare after hospital discharge | 4.21±4.40 | 3.32±3.85 | 0.13 | 4.10±4.28 | 3.18±3.98 | 0.19 | 4.52±4.28 | 3.37±4.12 | **0.04** | 3.38±4.09 | 4.27±4.16 | 3.79±4.25 | 0.64 | 4.06±4.21 | 3.81±4.22 | 0.69 | 4.40±4.25 | 3.29±4.04 | 3.81±4.39 | 0.22 |

1: Primary or no studies; 2: Secondary, including vocational; 3: University or further

**Supplementary material, Table 2. Bivariate analysis: variables associated with better experience scores**

| **Parameter** | **Overall IEXPAC experience score** | **Productive Interactions score (Factor 1)** | **New Relational Model score (Factor 2)** | **Patient Self-management score (Factor 3)** | **Continuity of healthcare after hospital discharge** |
| --- | --- | --- | --- | --- | --- |
| Age | - | - | Younger * | - | - |
| Gender | - | - | - | - | - |
| Educational level achieved | - | - | Higher * | - | - |
| Barthel Index >80 | - | - | - | - | - |
| Follow-up in a region different from home region (*vs*. same region) | - | - | - | - | - |
| Number of specialists visited in the past year | - | - | - | - | - |
| Follow-up by the same physician (*vs*. different) | Same physician | Same physician | - | Same physician | - |
| Follow-up by a nurse (*vs*. no nurse follow-up) | - | - | - | Follow-up by a nurse | - |
| Having help from others for healthcare (*vs*. self-care only) | - | - | - | - | Requiring help from others |
| Number of different medicines taken | - | - | - | - | - |
| Being treated with SC/IV drugs (*vs*. no SC/IV treatment) | - | - | - | - | - |

The table displays the values of the variables associated with better experience scores in the bivariate analysis (*P* < 0.05)

*Denotes a linear trend
